# Psychometric evaluation of the Swedish translation of the revised Cystic Fibrosis Questionnaire in adults

**DOI:** 10.1080/03009734.2016.1225871

**Published:** 2016-09-15

**Authors:** Jacek Hochwälder, Agneta Bergsten Brucefors, Lena Hjelte

**Affiliations:** a Department of Psychology, Mälardalens University, Eskilstuna, Sweden;; b Stockholm CF Centre, Karolinska University Hospital Huddinge, Stockholm, Sweden;; c Department of Public Health, Division of Intervention and Implementation Research, Karolinska Institute, Stockholm, Sweden

**Keywords:** Adults, cystic fibrosis, CFQ-R, health-related quality of life, psychometrics

## Abstract

**Aim:**

The CFQ-R is one of the most established disease-specific, health-related quality of life (HRQOL) measurements for patients with cystic fibrosis (CF). The aim was to evaluate the psychometric properties of the Swedish translation of CFQ-R in adults.

**Method:**

A total of 173 CF patients answered the CFQ-R. The CFQ-R was evaluated with regard to: (1) distributional properties; (2) reliability; and (3) construct validity.

**Results:**

The majority of scales were negatively skewed with ceiling effects. Eight of the 12 scales had satisfactory homogeneity; 10 of the 12 scales had satisfactory test–retest reliability. On many of the CFQ-R scales expected differences were observed when patients were divided regarding disease severity, nutritional status, age, and gender.

**Conclusion:**

Some weaknesses were detected, but overall the instrument has satisfactory psychometric properties.

## Introduction

Cystic fibrosis (CF) is the most frequent genetic, lethal disease in Caucasian populations (1). It affects mainly the respiratory tract but also the digestive and genito-urinary areas (1). One of the most often used clinical measures to assess the respiratory function is the forced expiratory volume in 1 second (FEV_1_), while a frequently used clinical measure of malnutrition is body mass index (BMI). However, these measures do not capture the full impact of the disease on the ability to function in various areas and on quality of life (2).

Various patient-reported, generic and disease-specific, health-related quality of life (HRQOL) measurements (3) are an important complement to the clinical measures. The generic measures can be used to compare persons with different diseases but are not sensitive to problems associated with a specific disease (3). The disease-specific measures target problems associated with a specific disease and have the advantage of being more sensitive to change as well as providing more information relevant for clinical interventions (3).

One of the most established specific measures for CF is the Cystic Fibrosis Questionnaire–Revised (CFQ-R) (3). The scale was originally developed in France (4) and revised in USA (3). There are three versions of the scale: 1) for children 6 to 13 years of age (CFQ-R-Child); 2) for parents to evaluate their children with CF (CFQ-R-Parent); and 3) for teenagers and adults (14 years or older) (CFQ-R-Teen/Adult) (3,4).

It is of fundamental importance that psychometric properties of a measure which is used in research and clinical practice are properly evaluated and reported (3,5). The CFQ-R-Teen/Adult has been translated into a number of languages, and the various translations have proved to have satisfactory psychometric properties (6). However, a Swedish translation of CFQ-R-Teen/Adult has yet not been psychometrically evaluated.

The general aim of the present study was to evaluate the psychometric properties of the Swedish translation of the CFQ-R-Teen/Adult in adults. More specifically, the aims were as follows. (1) To describe the distribution of CFQ-R. (2) To assess the reliability of the CFQ-R, in terms of homogeneity and test–retest reliability. (3) To assess construct validity of the CFQ-R, in terms of known-groups validity, based on the following four variables: *Disease severity*, where lower percentages of predicted forced expiratory volume in 1 second (FEV_1_%) were expected to be related to lower degrees of disease-specific HRQOL among CF patients (6,7). *Nutritional status*, where malnutrition was expected to be related to lower degrees of HRQOL (6,7). Because CF is a deteriorating medical condition, an increase in *age* was expected be associated with lower degrees of HRQOL (2,6,8,9). Because morbidity and mortality are higher among females than among male CF-patients, *gender* was related to HRQOL, where women were expected to be more strongly affected by CF than men (10–13).

## Method

### Procedure and participants

The participants were recruited from two CF centres in Sweden. During the monthly visit to their CF centre they completed the CFQ-R questionnaire, and their BMI and FEV_1_% were obtained. Of the 183 patients that were asked to participate in this study, 173 patients, 84 women and 89 men (*M*_Age_ = 30.80 years, SD_Age_ = 11.98 years, Min_Age_–Max_Age_ = 18–72 years; *M*_BMI_ = 22.25, SD_BMI_ = 3.42, Min_BMI_–Max_BMI_ = 16.91–35; *M*_FEV1%_ = 75.02, SD_FEV1%_ = 26.56, Min_FEV1%_–Max_FEV1%_ = 22–130) agreed to participate. To evaluate test–retest reliability of CFQ-R, 30 patients were asked to answer the CFQ-R on a second occasion, of which 26 patients, 15 women and 11 men (*M*_Age_ = 33.92 years, SD_Age_ = 12.92 years, Min_Age_–Max_Age_ = 19–72 years; *M*_BMI_ = 22.69, SD_BMI_ = 3.07, Min_BMI_–Max_BMI_ = 18.80–30.80; M_FEV1%_ = 71.08, SD_FEV1%_ = 24.79, Min_FEV1%_–Max_FEV1%_ = 25–111) agreed and participated once again after approximately 14 days. The study was approved by the research ethics committee of the Karolinska Institute of Stockholm.

### Measures

*Clinical variables*. The percentage of predicted forced expiratory volume in 1 second (FEV_1_%) and the body mass index (BMI = kg/m^2^) were noted.

*Demographical variables.* Gender and age were noted.

*CFQ-R-Teen/Adult*. The English version of CFQ-R-Teen/Adult (3,4) was translated independently by two researchers into Swedish. The two translations were compared, and some minor incongruities were resolved in order to agree on one single translation. This single translation was then back-translated to English by an authorized translator. The back-translated version was compared to the original English version of CFQ-R and was found to be almost identical (see Supplemental material available online).

The CFQ-R-Teen/Adult consists of 49 items measuring the following 12 domains: physical functioning (8 items); vitality (4 items); emotional functioning (5 items); eating disturbances (3 items); treatment burden (3 items); health perceptions (3 items); social functioning (6 items); body image (3 items); role limitations (4 items); weight (1 item); respiratory symptoms (6 items); and digestive symptoms (3 items). Each of the 49 questions are to be answered with reference to a time frame of the preceding two weeks. Answers are to be given on a 4-point Likert self-rating scale that includes frequency (always, often, sometimes, never), intensity (a great deal, somewhat, a little, not at all), and true–false (very true, somewhat true, somewhat false, very false). For each domain the answers are standardized to range from 0 to 100, where higher values indicate better HRQOL.

### Statistical analyses

All analyses were carried out using the SPSS program (14,15).

1. Distributional properties in form of arithmetic means, standard deviations, medians, quartiles, skewness (a measure of asymmetry of a distribution), and kurtosis (a measure of the extent to which observations cluster around the central point) were calculated for each sub-scale. A skewness value that is more than twice its standard error may be taken to indicate an asymmetric distribution, and a kurtosis value that is more than twice its standard error may be taken to indicate that, in comparison to the normal distribution, the distribution of scores is either more or less clustered around its central point (14,15). The floor effect was defined as a value ≤5 points on a scale, and the ceiling effect was defined as ≥95 points on a scale (3). For each scale, the percentage of respondents having a floor and a ceiling effect was reported.
2. Reliability was assessed in terms of homogeneity and test–retest reliability.
  - Homogeneity was calculated in terms of Cronbach’s alpha coefficient. Cronbach’s alpha coefficients ≥0.70 are usually considered as indicating satisfactory reliability (16).
  - Test–retest reliability was calculated using intra-class correlation coefficients (ICC), mixed model, absolute type, average measures (14,15). For a test–retest period of approximately two weeks ICC ≥0.80 can be considered as satisfactory (17).
3. Construct validity was evaluated in terms of known-groups validity based on disease severity, BMI, age, and gender. On the basis of FEV_1_%, patients were divided according to the international categorization into three severity groups (9): mildly impaired (FEV_1_ ≥71%), moderately impaired (FEV_1_ = 41%–70%), and severely impaired (FEV_1_ ≤40%). On the basis of BMI, patients were categorized into two groups (8): nourished (BMI ≥19) and malnourished (BMI <19). Based on age, patients were categorized into two groups (8); young adults (18–25 years), and adults (≥26 years). Based on gender, patients were categorized into men and women. Comparisons between sub-groups with regard to the 12 CFQ-R sub-scales were done using one-way MANOVAs for independent samples, followed up with ANOVAs for independent samples, and where more than two groups were compared the *F* test was followed up by Tamhane’s T2 *post hoc* tests. Effect sizes were calculated in terms of *η*^2^, where *η*^2^ = 0.01 (−0.059) represents a small effect, *η*^2^ = 0.06 (–0.139) a moderate effect, and *η*^2^ = 0.14 (or higher) represents a high effect (19), and in terms of Cohen’s *d*, where *d* = 0.20 (−0.49) represents a small effect, *d* = 0.50 (−0.79) a moderate effect, and *d* = 0.80 (or higher) represents a high effect (18).

## Results

### Descriptive statistics

The descriptive statistics for the CFQ-R are presented in Table 1. All scales—except one (treatment burden)—are significantly and negatively skewed. Three scales (physical functioning, eating disturbances, and body image) are leptokurtic (relative to the normal distribution, cluster more around the centre of the distribution and have thinner tails), and one (weight problems) is platycurtic (relative to the normal distribution, cluster less around the centre of the distribution and have thicker tails). For 5 of the 12 scales, rather small numbers of subjects had floor effects (0.60%–12.70%). There were ceiling effects (1.70%–57.80%) for all of the 12 scales.

**Table 1.** Descriptive statistics for the 12 CFQ-R scales.

Scales	*M*	SD	Median	1st Quartile	3rd Quartile	Skewness	Kurtosis	Floor effects: % with score ≤5	Ceiling effects: % with score ≥95
Physical functioning	82.27	22.92	91.67	75.00	100.00	−1.56*	1.75*	0.60	46.20
Vitality	59.92	20.40	66.67	41.67	75.00	−0.40*	−0.27	0.60	1.70
Emotional functioning	77.19	19.02	80.00	66.67	93.33	−0.87*	0.18	0	15.00
Eating disturbances	89.53	16.63	100.00	88.89	100.00	−2.17*	5.90*	0	57.80
Treatment burden	62.30	20.21	66.67	55.55	77.78	−0.09	−0.11	0	8.10
Health perceptions	63.20	23.46	66.67	44.44	77.78	−0.53*	−0.16	2.30	7.50
Social functioning	76.52	15.54	77.78	66.67	88.88	−0.83*	0.52	0	5.20
Body image	77.65	23.60	88.88	66.67	100.00	−1.31*	1.38*	1.70	27.70
Role limitations	83.67	19.88	91.67	75.00	100.00	−1.57*	2.21*	0	35.30
Weight problems	72.64	36.43	100.00	33.33	100.00	−0.96*	−0.57*	12.70	57.20
Respiratory symptoms	70.42	20.21	72.22	55.55	88.88	−0.54*	−0.44	0	6.90
Digestive symptoms	78.29	17.52	77.77	66.67	88.88	−0.84*	0.60	0	20.20

*P < .05.

### Reliability

Reliabilities are presented in Table 2. Four (treatment burden, social functioning, body image, and digestive symptoms) scales had Cronbach alpha coefficients below 0.70. Two (treatment burden and respiratory symptoms) scales had ICC below 0.80.

**Table 2.** Reliabilities for the 12 CFQ-R scales.

Scales	Cronbach alpha coefficient	Intra-class correlation coefficients (95% CI)
Physical functioning	0.93	0.96*** (0.91, 0.98)
Vitality	0.72	0.84*** (0.66, 0.93)
Emotional functioning	0.75	0.89*** (0.76, 0.95)
Eating disturbances	0.82	0.80*** (0.56, 0.91)
Treatment burden	0.56	0.71*** (0.36, 0.87)
Health perceptions	0.75	0.92*** (0.82, 0.96)
Social functioning	0.53	0.91*** (0.80, 0.96)
Body image	0.62	0.86*** (0.68, 0.94)
Role limitations	0.79	0.89*** (0.75, 0.95)
Weight problems	n.a.	0.88*** (0.74, 0.95)
Respiratory symptoms	0.85	0.74*** (0.42, 0.88)
Digestive symptoms	0.63	0.88*** (0.74, 0.95)

n.a. = not applicable (single-item scale).

***P < .001.

### Validity

*Comparison between the three severity groups*. As shown in Table 3, for 10 of the 12 scales, the mildly impaired had higher values (better HRQOL) than the moderately impaired, and the moderately impaired had higher values than the severely impaired. A one-way MANOVA indicated significant differences between the three severity groups with regard to the values on the 12 scales: Pillai’s trace = 0.51, *F*_24, 320_ = 4.55, *P <* .0001. Eight of the 12 one-way ANOVAs indicated significant differences between the three groups. For seven of these eight significant differences, Tamhane’s T2 *post hoc* test showed that the clearest differences—in the expected direction—were between the mildly and severely impaired. For all eight differences, *η*^2^ and Cohen’s *d* indicated either a medium or a strong effect.

**Table 3.** Comparisons between the three severity groups for the 12 CFQ-R scales.^a^

	Mildly impaired (*n* = 101)		Moderately impaired (*n* = 53)		Severely impaired (*n* = 19)				Cohen’s *d* (Tamhane’s T2 *post hoc* test)		
Scales	*M*	SD	*M*	SD	*M*	SD	*F*_2, 170_	*η*^2^^a^	Mildly versus Moderately impaired	Mildly versus Severely impaired	Moderately versus Severely impaired
Physical functioning	91.13	12.41	75.16	26.26	55.04	28.28	31.96***	**0.27**	0.78***	1.65***	0.74*
Vitality	62.13	19.70	58.49	21.89	52.19	18.39	2.11	0.02	0.17 (n.s.)	*0.52* (n.s.)	0.31 (n.s.)
Emotional functioning	78.22	17.98	77.11	20.78	71.92	19.45	0.87	0.01	0.06 (n.s.)	0.34 (n.s.)	0.26 (n.s.)
Eating disturbances	91.53	14.24	85.95	20.80	88.89	14.34	1.99	0.02	0.31 (n.s.)	0.11 (n.s.)	−0.16 (n.s.)
Treatment burden	66.34	19.34	58.91	19.92	50.30	20.07	6.50**	*0.07*	0.38 (n.s.)	0.81**	0.43 (n.s.)
Health perceptions	69.53	20.94	56.39	22.84	48.54	27.02	10.69***	*0.11*	0.60**	0.87**	0.31 (n.s.)
Social functioning	79.81	13.05	72.64	17.15	69.88	19.00	5.98**	*0.07*	0.47*	*0.61* (n.s.)	0.15 (n.s.)
Body image	82.40	20.38	75.89	22.41	57.31	31.48	10.24***	*0.11*	0.30 (n.s.)	0.95**	*0.68* (n.s.)
Role limitations	87.13	17.02	82.39	19.24	68.86	28.17	7.43***	*0.08*	0.26 (n.s.)	0.78*	*0.56* (n.s.)
Weight problems	81.18	31.42	66.04	37.83	45.61	41.88	9.79***	*0.10*	0.44*	0.96**	*0.51* (n.s.)
Respiratory symptoms	77.06	17.44	61.53	20.41	59.94	20.33	15.33***	**0.15**	0.82***	0.35**	0.08 (n.s.)
Digestive symptoms	76.68	17.25	80.71	17.72	80.12	18.36	1.04	0.01	−0.23 (n.s.)	−0.19 (n.s.)	0.02 (n.s.)

^a^Small effect size in ordinary type; medium in italics; large in bold.

n.s. = not significant (*P* > .05); *P < .05; **P < .01; ***P < .001.

*Comparison between nourished and malnourished*. As shown in Table 4, for 9 of the 12 scales, the nourished had higher values than the malnourished. A one-way MANOVA indicated significant differences: Pillai’s trace = 0.25, *F*_12, 160_ = 4.44, *P <* .0001. Four of the 12 one-way ANOVAs indicated significant differences between the two groups. The differences were observed on four scales relating to the body (physical functioning, eating disturbances, body image, and weight problems) and were in the expected direction. For three (eating disturbances, body image, and weight problems) of the four differences, *η*^2^ and Cohen’s *d* indicated either a medium or a strong effect.

**Table 4.** Comparisons between nourished and malnourished for the 12 CFQ-R scales.^a^

	Nourished (*n* = 152)		Malnourished (*n* = 21)				
Scales	*M*	SD	*M*	SD	*F*_1, 171_	*η^2^*	Cohen’s *d*
Physical functioning	83.66	22.50	72.22	23.95	4.69*	0.03	0.49
Vitality	60.31	20.29	57.14	21.45	0.44	0.00	0.15
Emotional functioning	77.15	18.71	77.46	21.65	0.00	0.00	−0.02
Eating disturbances	91.08	12.98	78.31	30.93	11.55***	*0.06*	*0.54*
Treatment burden	62.35	19.94	61.90	22.65	0.01	0.00	0.02
Health perceptions	63.45	23.22	61.38	25.73	0.14	0.00	0.08
Social functioning	77.08	14.86	72.49	19.76	1.62	0.01	0.26
Body image	80.99	20.28	53.44	31.35	29.29***	**0.15**	**1.04**
Role limitations	83.66	20.02	83.73	19.27	0.00	0.00	−0.00
Weight problems	77.63	32.73	36.51	42.04	27.08***	**0.14**	**1.09**
Respiratory symptoms	71.05	20.30	65.87	19.35	1.21	0.01	0.26
Digestive symptoms	78.22	17.53	78.84	17.88	0.02	0.00	−0.04

a Small effect size in ordinary type; medium in italics; large in bold.

*P < .05; ***P < .001.

*Comparison between young adults and adults*. As shown in Table 5, for 10 of the 12 scales, the young adults had higher values than the adults. A one-way MANOVA indicated significant differences: Pillai’s trace = 0.17, *F*_12, 160_ = 2.80, *P <* .002. Four of the 12 one-way ANOVAs indicated significant differences between the two groups. The differences were observed with regard to physical functioning, social functioning, body image, and respiratory symptoms, and on these four scales young adults had higher values compared to adults. For two (physical functioning and social functioning) of the four differences, *η*^2^ and Cohen’s *d* indicated a medium effect.

**Table 5.** Comparisons between young adults and adults for the 12 CFQ-R scales.^a^

	Young adults (*n* = 81)		Adults (*n* = 92)				
Scales	*M*	SD	*M*	SD	*F*_1, 171_	*η*^2^	Cohen’s *d*
Physical functioning	88.42	16.12	76.86	26.48	11.65***	*0.06*	*0.53*
Vitality	61.93	18.86	58.15	21.61	1.48	0.01	0.19
Emotional functioning	77.61	17.01	76.81	20.72	0.08	0.00	0.04
Eating disturbances	89.16	19.48	89.86	13.75	0.07	0.00	−0.04
Treatment burden	61.59	18.09	62.92	21.99	0.19	0.00	−0.07
Health perceptions	66.80	22.67	60.02	23.82	3.65	0.02	0.29
Social functioning	80.73	14.94	72.83	15.20	11.83***	*0.06*	*0.52*
Body image	81.62	21.53	74.15	24.88	4.39*	0.02	0.32
Role limitations	85.29	16.10	82.25	22.69	1.01	0.01	0.15
Weight problems	75.72	36.52	69.93	36.33	1.09	0.01	0.16
Respiratory symptoms	74.00	17.89	67.27	21.66	4.89*	0.03	0.34
Digestive symptoms	76.95	19.31	79.47	15.79	0.89	0.00	−0.14

a Small effect size in ordinary type; medium in italics; large in bold.

*P < .05; ***P < .001.

*Comparison between men and women*. As shown in Table 6, for 8 of the 12 scales, men had higher values than women. A one-way MANOVA indicated significant differences: Pillai’s trace = 0.14, *F*_12, 160_ = 2.18, *P <* .015. Two of the 12 one-way ANOVAs indicated significant differences between the two groups. The differences were observed with regard to body image and weight problems, and on these four scales women had higher values than men. For one (weight problems) of the two differences, *η*^2^ and Cohen’s *d* indicated a medium effect.

**Table 6.** Comparisons between men and women for the 12 CFQ-R scales.^a^

	Men (*n* = 89)		Women (*n* = 84)				
Scales	*M*	SD	*M*	SD	*F*_1, 171_	*η*^2^	Cohen’s *d*
Physical functioning	82.68	23.78	81.84	22.11	0.06	0.00	0.04
Vitality	61.61	19.97	58.13	20.82	1.26	0.01	0.17
Emotional functioning	79.03	18.92	75.24	19.04	1.72	0.01	0.20
Eating disturbances	89.89	14.47	89.15	18.73	0.08	0.00	0.04
Treatment burden	64.54	20.09	59.92	20.19	2.28	0.01	0.23
Health perceptions	63.29	24.24	63.09	22.76	0.00	0.00	0.01
Social functioning	77.15	15.57	75.86	15.58	0.30	0.00	0.08
Body image	72.66	26.86	82.94	18.29	8.56**	0.05	−0.45
Role limitations	83.61	19.72	83.73	20.16	0.00	0.00	−0.01
Weight problems	63.67	38.48	82.14	31.66	11.81***	*0.06*	*−0.52*
Respiratory symptoms	70.91	21.09	69.91	19.34	0.11	0.00	0.05
Digestive symptoms	79.28	17.42	77.25	17.67	0.58	0.00	0.12

a Small effect size in ordinary type; medium in italics; large in bold.

**P < .01; ***P < .001.

## Discussion

*The first aim* was to describe the 12 CFQ-R scales with regard to various distributional properties. The obtained means (and standard deviations) can be used in future assessments of specific Swedish CF patients to make their scores on the 12 CFQ-R scales more meaningful. For example, a patient’s values on the 12 scales may be compared to the means on each scale to locate the domain(s) in which the patient has distinctively low values (3). Similar to some previous findings (3,8), the majority of scales were negatively skewed with ceiling effects ranging from 1.70% (vitality) to 57.80% (eating disturbances). This finding can be interpreted to mean that a large proportion of the patients perceived themselves to possess rather good HRQOL in the 12 domains. Four scales were found to be leptokurtic, and one was found to be platycurtic.

*The second aim* was to assess reliability. Eight of the 12 scales had satisfactory homogeneity. Similar to some previous findings (3,8,19), four scales (treatment burden, social functioning, body image, and digestive symptoms) had somewhat low homogeneity (Cronbach alpha coefficient <0.70). Ten of the 12 scales had satisfactory test–retest reliability. Similar to some previous findings (4,6), two scales (treatment burden and respiratory symptoms) had somewhat low test–retest reliabilities (ICC <0.80).

*The third aim* was to assess construct validity. For the *three severity groups*, it was found that on 10 of the 12 scales the mildly impaired had better HRQOL than the moderately impaired and that the moderately impaired had in turn better HRQOL than the severely impaired. For 8 of the 12 scales the results were statistically significant in the expected direction. The non-significant differences were found for vitality, emotional functioning, eating disturbance, and digestive symptoms. Two studies (6,8) have shown significant differences in the expected directions for all scales except for digestive symptoms, and some studies (2,3,19) have shown significant differences on only some of the scales. For the *two BMI groups*, the nourished had higher values than the malnourished on 9 of the 12 scales, and for 4 of the 12 scales the results were statistically significant in the expected direction. The nourished had better HRQOL values on physical functioning, eating disturbances, body image, and weight problems, which partially overlaps with results obtained in some previous studies (2,6,8). All four domains are related to the physical aspect of body. For the *two age groups*, the young adults had higher values than the adults on 10 of the 12 scales, and for 4 of the 12 scales the results were statistically significant in the expected direction. As found in some previous studies (2,6), young adults had better HRQOL values on physical functioning and respiratory symptoms, which is expected because CF progresses with age. In addition, it was found that young adults also had higher values on social functioning and body image than adults. Finally, for *gender*, men had higher values than women on 8 of the 12 scales, but on 2 of the 12 scales results were statistically significant, although in the opposite direction to what was expected. As found in some previous studies (2,3,8), women had better HRQOL values on body image and weight problems than men. This may be explained with reference to our body-fixated society regarding thinness and low body-weight as more desirable for women than for men, even though this might have negative consequences for their health (2,3,6).

Once a measurement instrument has demonstrated reliability and validity, then it is of importance to assess if the observed changes on the instrument are clinically relevant or, in other words, the instrument’s minimal clinically important difference (MCID). Usually the MCID for CFQ-R has not been assessed (e.g. 2,8,9,19) except in one study made on two populations of patients with CF and chronic pseudomonas aeruginosa airway infection (20). Thus, the next step for future research should be to assess the MCID for CFQ-R in the Swedish adult CF population.

To conclude, the present evaluation of the Swedish translation of the CFQ-R in adults found some weaknesses for some scales (as has also been found in translations to other languages), but overall it can be considered that the CFQ-R possesses satisfactory psychometric properties. This translation and evaluation of the CFQ-R will contribute by making it possible to: (1) obtain additional important information about the HRQOL status of individual patients that attend Swedish CF centres for check-ups and treatment; (2) conduct research on CF in Sweden; and (3) compare Swedish CF patients with CF patients in other countries.
